# FEASIBILITY OF REMOTELY SUPERVISED ONLINE CHAIR YOGA INTERVENTION FOR OLDER ADULTS WITH DEMENTIA

**DOI:** 10.1093/geroni/igac059.269

**Published:** 2022-12-20

**Authors:** JuYoung Park, Marlysa Sullivan, Keri Heilman, Jayshree Surage, Hannah Levine, Lillian Hung, María Ortega

**Affiliations:** Florida Atlantic University College of Social Work and Criminal Justice, Boca Raton, Florida, United States; Maryland University of Integrative Health, Laurel, Maryland, United States; University of North Carolina School of Medicine Psychiatry, Chapel Hill, North Carolina, United States; Maryland University of Integrative Health, Laurel, Maryland, United States; Florida Atlantic University, Charles E. Schmidt College of Medicine, Boca Raton, Florida, United States; University of British Columbia, Vancouver, British Columbia, Canada; Florida Atlantic University Christine E. Lynn College of Nursing, Boca Raton, Florida, United States

## Abstract

This study assessed the feasibility of a remotely supervised online chair yoga (CY) intervention for older adults with dementia, exploring preliminary effects of CY on chronic pain, mobility, risk of falling, sleep disturbance, autonomic reactivity, cardiac rhythms (using IOM2 biofeedback device), and loneliness in this population. Using a one-group pretest/posttest design, a home-based CY intervention was delivered remotely to a group of 10 older adults with dementia who were socially isolated due to COVID-19. The online intervention was conducted twice weekly in 60-minute sessions for 8 weeks; data were collected virtually at baseline, mid-intervention, and post-intervention. Results indicated that online CY is a feasible approach for managing physical and psychological symptoms in older adults with dementia, based on retention (70%) and adherence (87.5%) with no injuries or other adverse events during the intervention. Senior-friendly videoconferencing should be available so that more older adults can gain access to the online intervention

